# The Effect of Low-Energy Laser-Driven Ultrashort Pulsed Electron Beam Irradiation on Erythropoiesis and Oxidative Stress in Rats

**DOI:** 10.3390/ijms23126692

**Published:** 2022-06-15

**Authors:** Gohar Tsakanova, Aida Avetisyan, Elena Karalova, Liana Abroyan, Lina Hakobyan, Anna Semerjyan, Naira Karalyan, Elina Arakelova, Violetta Ayvazyan, Lusine Matevosyan, Arpine Navasardyan, Anna Ayvazyan, Hakob Davtyan, Bagrat Grigoryan, Arsen Arakelyan, Zaven Karalyan

**Affiliations:** 1 Institute of Molecular Biology NAS RA, Yerevan 0014, Armenia; a.avetis@mail.ru (A.A.); karalovae@gmail.com (E.K.); pbzhikyan@yahoo.gr (L.A.); lina.hakobyan@gmail.com (L.H.); elinaa72@mail.ru (E.A.); viola_ay@yahoo.com (V.A.); lusine.matevosyan@inbox.ru (L.M.); aarakelyan@sci.am (A.A.); zkaralyan@yahoo.com (Z.K.); 2 CANDLE Synchrotron Research Institute, Yerevan 0040, Armenia; arpinenavasardyan@gmail.com (A.N.); anayvazyan95@gmail.com (A.A.); davtyan@asls.candle.am (H.D.); grigory@asls.candle.am (B.G.); 3 Experimental Laboratory, Yerevan State Medical University after Mkhitar Heratsi, Yerevan 0025, Armenia; 4 Department of Medical Biology and Genetics, Yerevan State Medical University after Mkhitar Heratsi, Yerevan 0025, Armenia; annasemerjian77@gmail.com; 5 Department of Pathological Anatomy and Clinical Morphology, Yerevan State Medical University after Mkhitar Heratsi, Yerevan 0025, Armenia; naira_karalyan@mail.ru

**Keywords:** ultrashort pulsed electron beam irradiation, oxidative stress, two-photon imaging, erythropoiesis, erythrocytes, *Wistar* rats

## Abstract

Anemia is a commonly observed consequence of whole-body exposure to a dose of X-ray or gamma irradiation of the order of the mean lethal dose in mammals, and it is an important factor for the determination of the survival of animals. The aim of this study was to unravel the effect of laser-driven ultrashort pulsed electron beam (UPEB) irradiation on the process of erythropoiesis and the redox state in the organism. *Wistar* rats were exposed to laser-driven UPEB irradiation, after which the level of oxidative stress and the activities of different antioxidant enzymes, as well as blood smears, bone marrow imprints and sections, erythroblastic islets, hemoglobin and hematocrit, hepatic iron, DNA, and erythropoietin levels, were assessed on the 1st, 3rd, 7th, 14th, and 28th days after irradiation. Despite the fact that laser-driven UPEB irradiation requires quite low doses and repetition rates to achieve the LD_50_ in rats, our findings suggest that whole-body exposure with this new type of irradiation causes relatively mild anemia in rats, with subsequent fast recovery up to the 28th day. Moreover, this novel type of irradiation causes highly intense processes of oxidative stress, which, despite being relatively extinguished, did not reach the physiologically stable level even at the 28th day after irradiation due to the violations in the antioxidant system of the organism.

## 1. Introduction

With the recent advances in accelerator technologies, new types of electron beam linear accelerators were developed based on ultrashort pulsed laser technologies, an emerging new direction in radiation biology and radiobiomedicine. Such unique laser-driven ultrashort pulsed electron beams (UPEBs) are capable of providing ultrashort picosecond pulses reaching repetition rates as low as just several Hertz and high doses as much as several orders of magnitude higher (10^11^ Gy/min) than conventional beams [1,2,3,4,5,6,7,8].

Such an approach to delivering ultrashort sub-picosecond pulses makes it possible to achieve a significant reduction in the interaction time, thereby mitigating the side effects in biological samples and opening up new and more efficient perspectives for radiation therapy. Thus, it was shown that very high energy electron beams are able to target deep tumors with higher radiation doses delivered to the tumor versus normal tissues, thereby providing potential clinical benefits by targeting deep tumors and improving the tumor-to-healthy-tissue ratio [9,10]. These features allow this new modality to be considered in future long-term biomedical studies as a promising, more effective, and less harmful radiotherapeutic approach for cancer treatment.

In the case of electron beam irradiation, samples absorb energy from the electrons, causing various chemical and biological alterations [11]. The absorption of ionizing radiation by living cells can directly disrupt atomic structures, producing chemical and biological changes. It can also act indirectly through radiolysis of water, thereby generating reactive oxygen species (ROS), which in turn trigger harmful oxidative processes [12]. In normal physiological conditions, the antioxidant system of the organism is highly efficient in the neutralization of generated ROS. However, the excess of ROS generated by ionizing radiation, as well as radiation-induced alterations in the antioxidant system itself, result in the failure of the organism to maintain the physiological redox balance. All this together leads to oxidative stress, which in turn may lead to deleterious effects on the organism [12,13,14].

Being responsible for the transportation of oxygen from the lungs throughout the body, among all the cells in the organism, the erythroid cells circulating in the blood stream, red blood cells (RBCs), are among the first cells in the organism affected by ROS [15,16]. It is not only the erythroid cells circulating in the blood stream that may be vulnerable to the effect of radiation; the radiation may cause harmful consequences in the process of erythropoiesis as whole [17,18]. Particularly, it has been shown that ROS play a crucial role in the deleterious effect of radiation on the process of erythropoiesis [19,20,21].

In fact, erythropoiesis is a highly coordinated and complex differentiation process originating in the bone marrow from a multipotent stem cell and terminating in a mature, enucleated erythrocyte with a characteristic biconcave shape [22,23,24]. Erythroid cells are released from the bone marrow and spleen as reticulocytes that rapidly (within 1 to 2 days) differentiate into mature erythrocytes in blood [25,26,27]. Erythropoiesis is initiated when the blood supply of oxygen necessary for normal tissue function is altered. The response to such a reduction in oxygen delivery occurs mainly in the kidney, which produces a humoral cytokine, erythropoietin, to target erythroid progenitor cells in the bone marrow [22,28].

Although the unique features of laser-driven UPEB provide promising prospects for the possible mitigation of the harmful effects of radiation, investigations into the effect of this new type of irradiation are only at the initial stage, and there are very limited data about their radiobiological effect [1,2,29,30,31,32]. Particularly, there are no systematic data for the processes of damage and restoration of bone marrow hematopoiesis during laser-driven UPEB irradiation. Thus, the aim of this study was to unravel and understand the effect of laser-driven UPEB irradiation with regard to the process of erythropoiesis and the redox state in the organism.

## 2. Results

### 2.1. Two-Photon Fluorescence Imaging of Oxidative Stress in Living Erythrocytes

**Figure 1:**
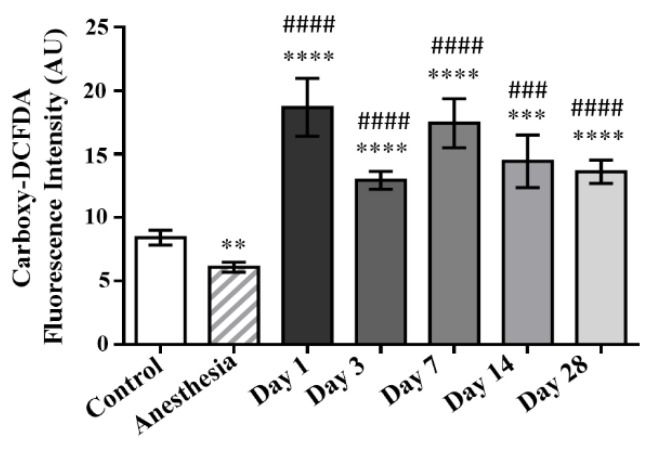
The mean intensities of the carboxy-DCFDA two-photon fluorescence of RBCs in control and irradiated animals measured on the 1st, 3rd, 7th, 14th, and 28th days after irradiation with the laser-driven UPEB. ** *p* < 0.01; *** *p* < 0.001; **** *p* < 0.0001; ### *p* < 0.05; #### *p* < 0.01 (* compared to control; # compared to anesthesia).

**Figure 2:**
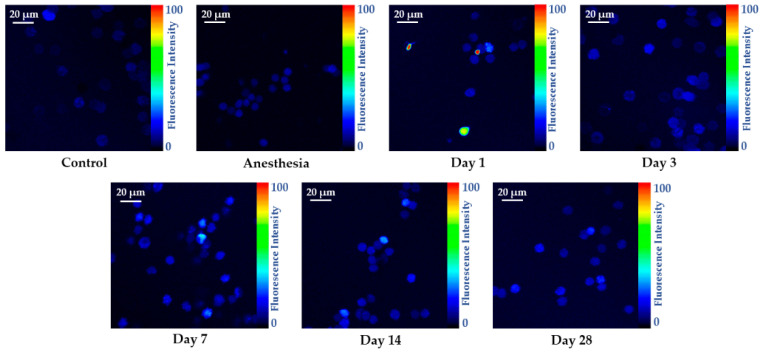
Representative two-photon fluorescence intensity images of RBCs from the control and irradiated animals measured on the 1st, 3rd, 7th, 14th, and 28th days after irradiation with the laser-driven UPEB.

The results of the two-photon fluorescence imaging of oxidative stress in living erythrocytes using the control rats and those exposed to the laser-driven UPEB irradiation are presented in Figure 1, and the representative two-photon fluorescence intensity images of RBCs are shown in Figure 2. The irradiation of the animals with the laser-driven UPEB initiated highly significant, intense oxidative processes on day 1 after irradiation, with a decrease following on day 3 (yet remaining significantly higher compared to control). Interestingly, a resumption of the significantly intense oxidative processes was registered on day 7. Despite the relative mitigation of oxidative processes on day 14 and then on day 28 after irradiation, the animals were not able to achieve either full, or partial recovery from the outbreak of oxidative stress initiated by the laser-driven UPEB irradiation.

### 2.2. The Effect of Laser-Driven UPEB Irradiation on the Antioxidant System

**Figure 3:**
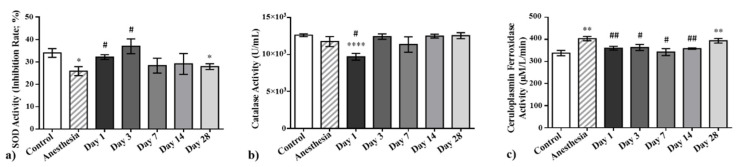
The activities of superoxide dismutase (**a**) and catalase (**b**), as well as the ferroxidase activity of ceruloplasmin (**c**), in the plasma samples of control and irradiated animals measured on the 1st, 3rd, 7th, 14th, and 28th days after irradiation with the laser-driven UPEB. * *p* < 0.05; ** *p* < 0.01; **** *p* < 0.0001; # *p* < 0.05; ## *p* < 0.01 (* compared to control; # compared to anesthesia).

The results for the activities of superoxide dismutase and catalase in erythrocyte hemolysates, as well as the ferroxidase activity of ceruloplasmin, in the plasma samples of the control rats and those exposed to the laser-driven UPEB irradiation are presented in Figure 3. Regarding superoxide dismutase, a significant and progressive increase in its activity was observed on day 1 and day 3 after irradiation and a sharp decrease on day 7, after which it remained on the same level up to day 28 after irradiation.

In contrast, a significant decrease in the activity of catalase was revealed on day 1 and a subsequent sharp recovery already on day 3, even achieving the control levels. This recovery was kept relatively stable up to day 28 after irradiation.

Finally, the ferroxidase activity of ceruloplasmin was significantly decreased on day 1 and subsequently maintained the same levels up to day 14. However, the ferroxidase activity of ceruloplasmin demonstrated an increase on day 28 after the laser-driven UPEB irradiation.

### 2.3. The Effect of Laser-Driven UPEB Irradiation on the RBC Characteristics

**Figure 4:**
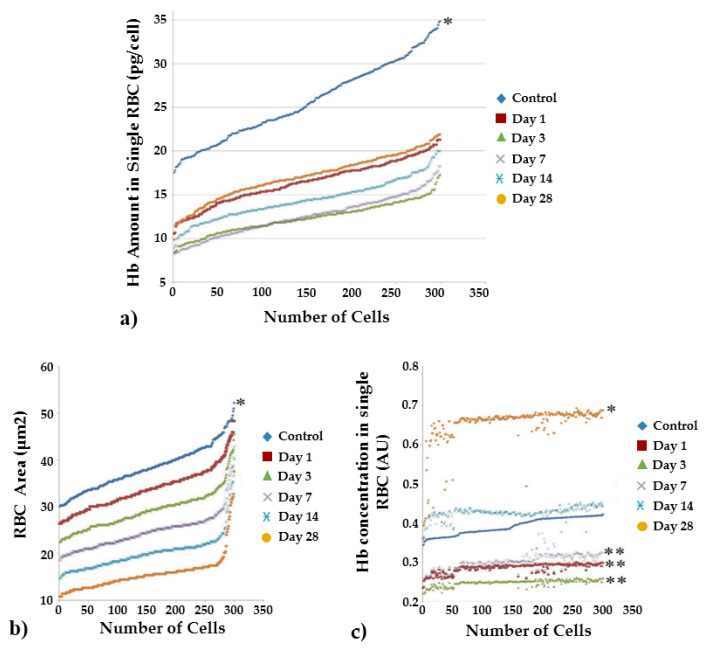
Characteristics of RBC samples from control and irradiated animals measured on the 1st, 3rd, 7th, 14th, and 28th days after irradiation with the laser-driven UPEB. (**a**) Hb amount in single RBC; (**b**) RBC size; (**c**) Hb concentration in single RBC. Each point represents the average of three RBCs. * *p* < 0.05; ** *p* < 0.01 (*compared to anesthesia).

To define the RBC characteristics, hematologic analysis was conducted to reveal the RBC size and the Hb amount and concentration in the peripheral blood smears of the control rats and those exposed to the laser-driven UPEB irradiation (Figure 4).

A total of seven animals were investigated for each point. The scatter plot in Figure 4a represents the Hb amounts for single RBCs in averaged populations. To facilitate understanding, the cells are arranged in terms of an increasing amount of hemoglobin. Each point corresponds to the average value of three RBCs. As seen in Figure 4a, there was a pronounced decrease in the hemoglobin content in RBCs after irradiation with the laser-driven UPEB. Thus, the Hb levels in RBCs began to increase only from days 14–28 after irradiation.

The results for the effects of the laser-driven UPEB on single RBC sizes in the control rats and those exposed to the laser-driven UPEB irradiation are presented in Figure 4b. There was a continuous decrease in the sizes of erythrocytes starting from day 1 after irradiation and until the end of the study (i.e., day 28 after irradiation), resulting in the formation of microcytosis.

The results for the effects of the laser-driven UPEB on single RBC Hb concentrations in the control rats and those exposed to the laser-driven UPEB irradiation are presented in Figure 4c. Furthermore, a decrease in the concentration of Hb in single RBCs was revealed on days 1, 3 and 7 after irradiation compared to control. Subsequently, the Hb concentration generally reached the same level on day 14 after irradiation as the control animals, and significantly exceeded the control level on day 28 after irradiation.

### 2.4. Hemolysis of Erythrocytes

**Figure 5:**
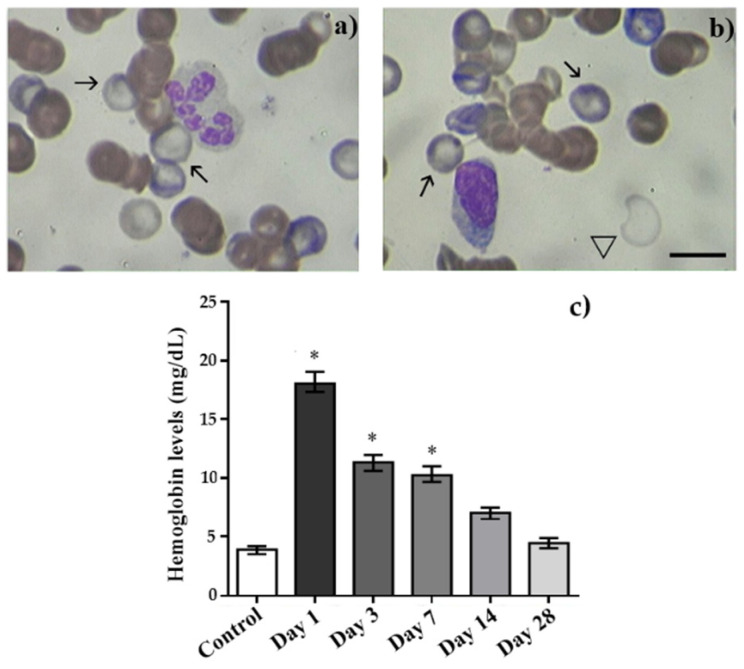
Evidence of hemolysis in peripheral blood samples of control and irradiated animals measured on the 1st, 3rd, 7th, 14th, and 28th day after irradiation with the laser-driven UPEB. (**a**) Ghost RBC (arrows) in rat blood smears on 1st day after irradiation; (**b**) ghost RBCs (arrows); membrane of RBC (triangle); (**c**) hemoglobin levels in plasma samples of control and irradiated animals. * *p* < 0.05 (compared to control). Scale bar is 10 μm.

In this part of the work, we studied the effect of laser-driven UPEB on hemolysis. The analyses of the hemolysis in the RBCs in the peripheral blood smears of control rats and those exposed to the laser-driven UPEB irradiation are presented in Figure 5. The microscopic investigation revealed the occurrence of both ghost RBCs (arrows in Figure 5a,b) and empty membranes of RBCs (triangle in Figure 5b). These phenomena mainly occurred only on day 1 after irradiation, and very few numbers of such cells appeared on day 3 after irradiation. Furthermore, as seen in Figure 5c, our investigations revealed significantly increased levels of plasma hemoglobin associated with increased intravascular erythrocyte destruction; i.e., hemolysis. Finally, the microscopic investigations of the liver sections stained by Perl’s Prussian blue did not reveal any significant amounts of iron (hemosiderin) deposition in the tissues of any investigated animals in any of the groups.

### 2.5. Regeneration of Bone Marrow Hematopoietic Tissue

**Figure 6:**
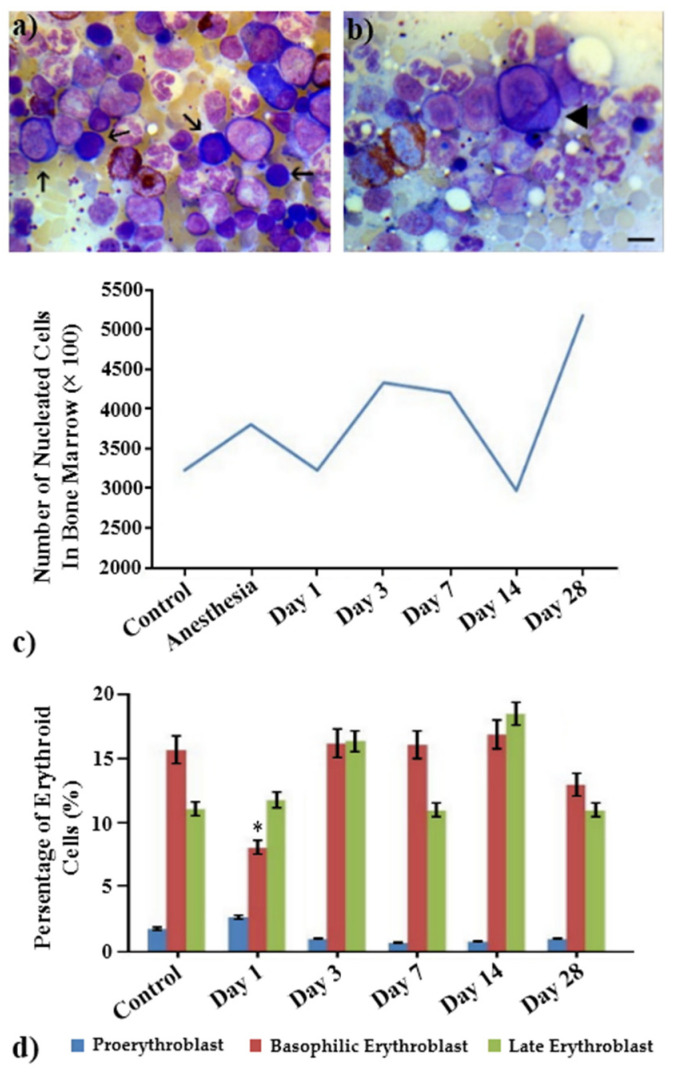
Erythroid cell populations in bone marrow samples of control and irradiated animals measured on the 1st, 3rd, 7th, 14th, and 28th days after irradiation with the laser-driven UPEB. (**a**,**b**) Representative images of bone marrow samples of control and irradiated animals (the arrows in (**a**) point to basophilic erythroblasts and the triangle in (**b**) points to the presence of proerythroblast). (**c**) Total number and (**d**) populations of nucleated erythroid cells in the bone marrow samples of control and irradiated animals. * *p* < 0.05 (compared to control). Scale bar is 10 μm.

The investigation of the bone marrow smears of the studied animal groups revealed a significant decrease in the population of basophilic erythroblasts starting from the early stage of irradiation compared to control (Figure 6a), while maintaining proerythroblasts (Figure 6b). It should be noted that no decrease in the total number of nucleated cells in the bone marrow appeared, although there was a tendency for a decrease in the nucleated cells on days 1 and 14 after irradiation (Figure 6c). As seen in Figure 6d, the most pronounced change in the nuclear cells of the erythroid population of the bone marrow was the significant decrease in the cells of the population of basophilic erythroblasts (Figure 6d).

**Figure 7:**
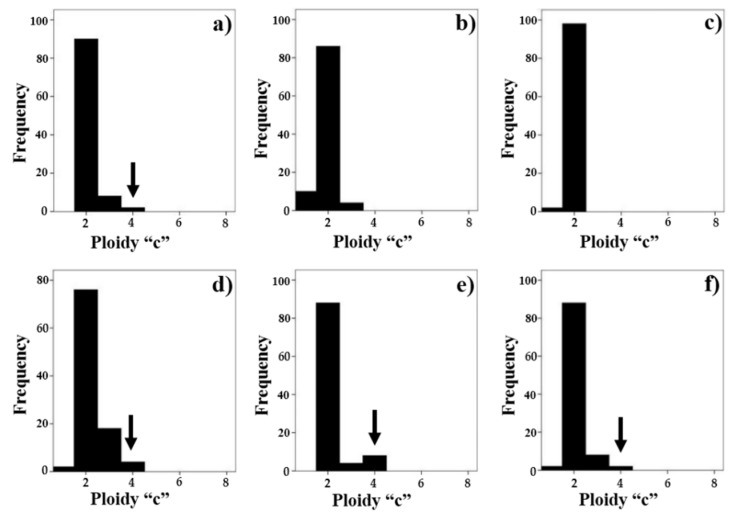
Distribution of the nuclei of nucleated erythroid cells (by ploidy classes) in the bone marrow samples of control (**a**) and irradiated animals on day 1 (**b**), day 3 (**c**), day 7 (**d**), day 14 (**e**), and day 28 (**f**) after irradiation.

Figure 7 shows the dynamics of the changes in the distribution of erythroblast nuclei by ploidy classes. Thus, in control animals (Figure 7a), the main population of diploid cells was noticeable (these were late erythroblasts and some of the basophilic erythroblasts), and a small number of cells in phase C of the mitotic cycle and tetraploid cells (all these cells are basophilic erythroblasts (arrows)) were revealed. Immediately after irradiation, on days 1–3 (Figure 7b,c), tetraploid nuclei completely disappeared, which indicated a decrease in basophilic erythroblasts due to damage or as compensation for the loss of RBCs. Nevertheless, already by the 7th to 14th days (Figure 7d,e), the population of basophilic erythroblasts in the bone marrow of irradiated animals was restored (arrows), and by the 28th day it almost did not differ from the control (Figure 7f; tetraploid basophilic erythroblasts (arrows)).

### 2.6. Alterations in Erythroblastic Islets

**Figure 8:**
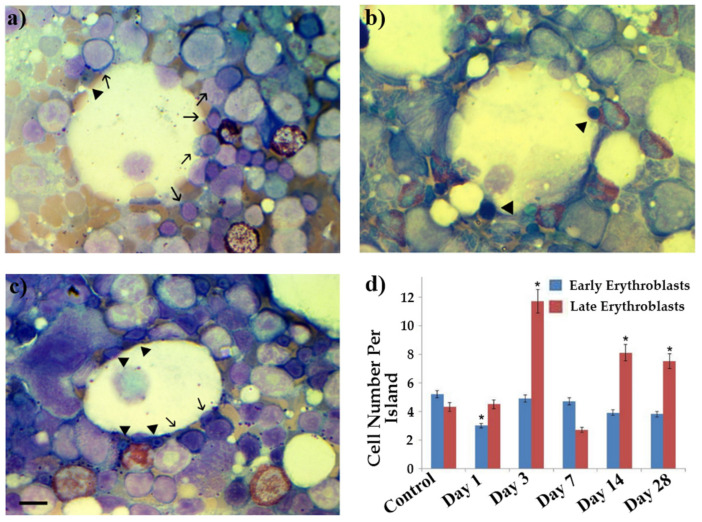
Erythroblastic islets and related erythroid cells. (**a**) Erythroblastic islets in healthy (anesthesia) rats’ bone marrow (early erythroblasts shown by arrows, late erythroblasts shown by triangles). (**b**) Erythroblastic islets in irradiated rats’ bone marrow on 1st day after irradiation. (**c**) Erythroblastic islets in irradiated rats’ bone marrow on 28th day after irradiation. (**d**) Changes in population of erythroid cells in erythroblastic islets in control and irradiated rats. * *p* < 0.05–*p* < 0.001 (compared to control). Scale bar is 10 μm.

To identify the causes of changes in the erythroid population of peripheral blood and bone marrow smears under the influence of an electron beam, we studied the erythroblastic islets in the bone marrow of rats. The data are shown in Figure 8.

In the control pool (Figure 8a,d) for the erythroid cells in the erythroblastic islets of rats, most of the cells were early blasts (proerythroblasts rarely, but mainly basophilic erythroblasts) and slightly more than 40% were late blasts (polychromatophilic and oxyphilic erythroblasts). Immediately after irradiation, the population of basophilic erythroblasts in the erythroblastic islets of rats sharply decreased (by almost two times; Figure 8b,d). The number of late erythroblasts remained unchanged. Then, the number of late erythroblasts greatly increased, far outnumbering the early cells. On the 28th day after irradiation (Figure 8c,d), the difference slightly leveled off; however, late cells continued to make up the majority of erythroblasts in the erythroblastic islets.

### 2.7. The Effect of Laser-Driven UPEB Irradiation on Plasma Levels of Erythropoietin

**Figure 9:**
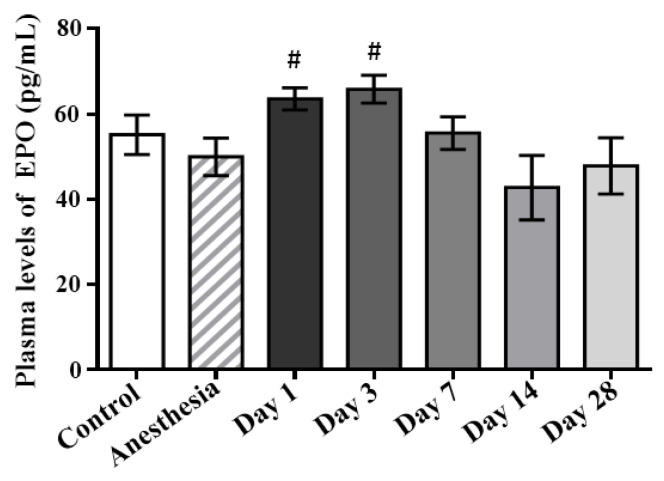
Plasma erythropoietin levels in control and irradiated rats measured on the 1st, 3rd, 7th, 14th, and 28th days after irradiation. # *p* < 0.05 (compared to anesthesia).

The concentrations of erythropoietin in the plasma samples of the control rats and those exposed to the laser-driven UPEB irradiation (Figure 9) were also measured. According to the results, a continuous and significant increase in the plasma erythropoietin levels was found on day 1 and day 3, with a subsequent decrease on day 14 to the control levels. Despite the plasma levels of erythropoietin demonstrating a subsequent decrease (albeit statistically non-significant), recovery up to the control levels already appeared on day 28 after irradiation.

## 3. Discussion

It is well-known that anemia is a commonly observed consequence of whole-body exposure to a dose of X-ray or gamma irradiation of the order of the mean lethal dose in mammals, and it is an important factor for the determination of the survival of the animals [33,34]. We described the anemia phenomenon in laser-driven UPEB irradiated rats in our recent report [35]. Given the large amount of continuously and rapidly proliferating cells, the hematopoietic system is considered one of the most radiosensitive compartments in the organism [36]. The effect of radiation (either γ- or X-rays) on the hematopoietic system depends on a number of factors: (1) the radiosensitivity of precursor cells; (2) the stage of the cell cycle and the lifespan of various cells; and (3) the ability of the hematopoietic tissue to regenerate after radiation injury [36,37]. The radiation exposure has been shown to affect different cell types in the hematopoietic system, including the immune response of T lymphocytes, depletion of T and B cells [38], violation in the differentiation of bone marrow hematopoietic progenitor cells [39], and hematologic alterations of CD34+ cells, early blast cells, macrophages [36], etc.

Gamma or X-ray irradiation in sublethal doses causes cellular depletion of the bone marrow in experimental animals, including rats. In rats, such an effect is usually observed immediately after irradiation, reaching its maximum at 1–3 days after irradiation. Hematopoietic cell injury is the primary cause of death after X-ray and gamma radiation [40,41].

Recently, we demonstrated that the use of laser-driven UPEBs greatly mitigates and reduces the effects of whole-body radiation of the animals [42]. Specifically, in that study we did not detect any complete or even partial long-term emptying of hematopoietic cells. In the bone marrow, only a 10–15 percent decrease in the total cell count was revealed [42]. This phenomenon was of a short-term nature and completely disappeared from 3 days after irradiation. In the population of hematopoietic cells, a significant decrease in basophilic erythroblasts was noticeable (just after irradiation), probably due to their ability to divide [43]. It is well-known that radiation is very effective for any actively proliferating cell system [44,45].

Hence, it is necessary to answer the question why the restoration of the population of erythroblasts in the bone marrow begins 7 days after irradiation with laser-driven UPEB. The fact is that the full cycle of maturation of erythropoietic cells in the bone marrow from proerythroblast to erythrocyte takes about 5 days [46,47].

We can conclude that there is today a lack of knowledge about the responses of erythroid progenitors and precursors to radiation injury [48]. Erythroblastic islets in bone marrow smears represent associations of macrophage cells and erythroid cells at various stages of differentiation. Classification of erythroblastic islets by maturity classes is based on morphological evaluation of the erythroid cells in the erythroblastic islets, starting from the proerythroblasts and basophilic erythroblasts and eventually considering the last division of oxyphilic erythroblasts [49]. Given the qualitative changes in the population of bone marrow cells, we can assume their compensatory value. However, despite the preservation of the functional activity of islet macrophages, such changes may be caused by their damage during irradiation.

In this study, we revealed a significant decrease in the concentration of hemoglobin, both in the general population of erythrocytes and in individual erythrocytes after the whole-body irradiation with laser-driven UPEB. This phenomenon was detected immediately after the irradiation (1st−3rd days), after which the recovery process began, and by the 14th day after irradiation the hemoglobin concentration reached levels comparable to the control and, subsequently, significantly exceeded the control levels on the 28th day. A low mean corpuscular hemoglobin concentration (MCHC) showed that the RBCs did not have enough hemoglobin, and the most common cause of low MCHC is anemia. This phenomenon is undoubtedly a reactionary response of hematopoietic tissue to hemolysis and subsequent recovery.

The biological effects of different types of irradiation on erythrocytes is mainly determined by the effect on the functional state of the membranes of RBCs. Usually, mature non-nucleated erythrocytes are relatively resistant to radiation damage. However, it has been shown that high doses of gamma- or X-rays can cause different pathological changes in RBCs [50,51,52]. It is therefore important to conduct research for the identification of the hemolysis in the peripheral blood of irradiated animals [34,53,54,55,56]. In the current study, we revealed the hemolysis of mature erythroid cells (containing hemoglobin), which led to the increase in the content of extracellular hemoglobin in the blood plasma. When irradiated with the laser-driven UPEB, hemolysis was observed only in the period immediately after irradiation. Then, there was a gradual but consistent decrease in the content of extracellular hemoglobin in the blood plasma, which can be explained by reactive recovery processes after the disappearance of the cause of hemolysis.

Levels of iron in tissue that are too low to be detected by Perl’s Prussian blue indicate insignificant and/or short-term hemolysis during irradiation [57]. It is well-known that when intravascular hemolysis occurs it is often possible to observe erythrocyte ghosts. This is the result of damage in the RBC membrane, causing true in vivo intravascular hemolysis with consequent hemoglobinemia. Despite the revelation of hemolysis and the presence of free hemoglobin in the plasma in the early stages after irradiation, it should be recognized that the anemia in the rats caused by the laser-driven UPEB was relatively mild. Only very low levels of positive reactions were detected (compared with healthy controls), and not all animals in each irradiated group were positive in the Perl’s Prussian blue reaction. This indicated a low level of serum ferritin; i.e., roughly or less than 1000 ng/mL [58]. It is worth to mentioning here that, in the case of irradiation of rats with gamma irradiation, a more pronounced degree of maximum aplasia in the hematopoietic tissue has been reported previously [42].

Thus, we identified damage to erythrocytes caused by the laser-driven UPEB, most likely associated with the disruption of the cell membrane integrity. The reason for this phenomenon was most likely the processes associated with oxidative stress. Indeed, we found an increase in the oxidative stress processes immediately after irradiation with laser-driven UPEB.

The damage to membrane proteins was shown to be responsible for the impaired RBC deformability associated with oxidative stress. This role of protein damage in producing impaired deformability is consistent with findings concerning a dominant role for the membrane cytoskeleton in regulating RBC deformability [59]. As shown by Mohanty et al. [60], although mature RBCs do not have one of the main types of superoxide dismutase (SOD2), its deficiency or malfunction during erythroid development results in an increase in oxidative stress in RBCs.

To understand to what extent the laser-driven UPEB irradiation caused alterations in the antioxidant systems of the animals, the activities of superoxide dismutase and catalase, as well as the ferroxidase activity of ceruloplasmin, were studied. As is obvious from Figure 3, the laser-driven UPEB irradiation resulted in the malfunction of the antioxidant enzymes catalase and ceruloplasmin on the first day after irradiation. Simultaneously, hyperactivation of SOD was registered, which can be considered a compensatory mechanism to maintain the redox balance and the homeostasis of the organism. However, the increased oxidative stress shown in Figure 1 and Figure 2 provides evidence that even the switch in compensatory mechanisms in the organism was not able to fully restore the redox balance and return the organism to the initial conditions, despite the full recovery of the activities of the SOD, catalase, and ceruloplasmin appearing on or up to the 28th day after irradiation.

With this in mind, the short-term rise in the level of erythropoietin in the plasma samples of rats detected in the current study immediately after irradiation may have a dual role. One role could be the stimulation of erythropoiesis, which was possibly the main reason for the insignificant anemia revealed, although this is unlikely. On the other hand, erythropoietin could have acted as an antioxidant to mitigate and reduce oxidative stress induced by the laser-driven UPEB irradiation [61].

It was previously reported that the regulatory mechanisms in human RBCs are more effective than in rat RBCs, allowing the cells to adapt better to different environments [62]. This may be because of the significant difference in the deformability of RBCs between rats and humans [62], which in turn may depend on the size of the RBCs. Thus, it is known that rat RBCs are smaller in size compared to human RBCs [62], and it can be considered that, in contrast to larger human RBCs, rat RBCs with smaller sizes do not need to go through significant deformations to enter microcirculation, thereby showing lower deformability than human RBCs [62,63,64,65]. Moreover, due to the lower surface charge, human RBCs demonstrate a higher percentage of aggregation and faster aggregation than rat RBCs [62]. The comparative analysis showed that rat RBCs have a higher level of fragility than human RBCs [62]. Taking into account all these differences found previously, it can be assumed that in the case of human irradiation with laser-driven UPEBs, the recovery processes should take place even faster. However, taking into account that animal experiments do not necessarily mimic the effects in human translational studies, further experiments need to be undertaken.

## 4. Materials and Methods

### 4.1. Animals

In the experimental design, forty-two male albino Wistar rats with weights of 180–200 g were used. Controlled colony conditions were provided for the housing and care of the animals, with the maintenance of 12 h reverse light–dark cycles in a temperature-regulated (22 °C) animal facility. A standard laboratory chow diet (B&K Universal) was maintained for the feeding of the animals, with ad libitum accessibility to food and water. The experiment was designed with seven randomly divided animal groups each consisting of six rats: control (non-irradiated animals), anesthesia (non-irradiated anesthetized animals), day 1, day 3, day 7, day 14, and day 28 (irradiated animals from which the samples were collected on the mornings of the 1st, 3rd, 7th, 14th, and 28th days after radiation exposure).

The animal protocol was approved by the Animal Ethical Committee of the Institute of Molecular Biology NAS RA, and the experiments were carried out in accordance with its relevant guidelines and regulations. All the rats except those in the control group were anesthetized with an intramuscular administration of 20 mg/kg aminazine (chlorpromazine hydrochloride, Sigma-Aldrich, Taufkirchen, Germany). Adequate depth for the anesthesia was ensured by testing the pedal withdrawal and palpebral reflexes. All the rats were euthanized by carbon dioxide overdose, following which blood samples were collected from the carotid artery and appropriate tissue samples.

### 4.2. Irradiation

Whole-body irradiation was delivered at 10:00–11:00 AM with a dose of 2 Gy and a repetition rate of 2 Hz, as described previously [42]. As an irradiation source, the laser-driven UPEB from the Advanced Research Electron Accelerator Laboratory (AREAL) facility was used, as developed at the Center for the Advancement of Natural Discoveries using Light Emission (CANDLE) Synchrotron Research Institute, Yerevan, Armenia. The details and procedure for the radiation treatment, the characteristics of the AREAL accelerator, the dose calibration measurements, and the uniformity quality assurance have been described previously [1,2,32,35,42,66].

The facility enables stable operations using an electron beam with well-formed and reproducible characterizing parameters. All machine parameters were measured and compared to previous ones before every experiment. The radiation treatment was performed using the AREAL laser-generated electron beam with a beam charge of 30 pC, electron energy of 3.6 MeV, pulse duration of 450 fs, pulse repetition rate of 2 Hz, beam spot of 15 mm, normalized transverse emittance of less than 0.5 mm-mrad, and a root-mean-square (RMS) energy spread of less than 1.5%. The electron bunch was generated using a UV laser pulse illuminating a copper photocathode. The pulse UV energy was about 350 µJ, the repetition rate was 2 Hz, the energy stability was less than 0.1%, the beam divergence was less than 0.1 mrad, and the spot size at the cathode was 2 mm. Online dose information was assessed using a Faraday cup as a dose-rate-independent absolute dosimeter.

### 4.3. Blood Smears, Giemsa Staining, and Blood Cell Analysis

Fresh blood collected from the carotid artery of irradiated and control rats was used when preparing the blood smears with routine methods. For the analysis of erythroid cells, slides were fixed in pure methanol and stained with Giemsa modified solution (azure B/azure II, eosin, and methylene blue), according to the manufacturer’s protocol (Sigma-Aldrich, Taufkirchen, Germany). White blood cells were examined under a light microscope at 1250× magnification in a random sequence. The evaluation of cells and their sizes based on morphologic characteristics was undertaken as described previously [67].

### 4.4. Bone Marrow Imprints

Bone marrow samples were obtained from the humerus, as described previously [68,69]. A standard cell counter was used to count the absolute number of nucleated cells. Morphological analysis of the bone marrow cells was performed on smears obtained from the contralateral humerus, and differential counts were performed according to the standard morphologic criteria for rats, as reported previously [67,70,71].

For cell analysis, slides were fixed in pure methanol and stained with modified Giemsa solution (azure B/azure II, eosin, and methylene blue), according to the manufacturer’s protocol (Sigma-Aldrich, Taufkirchen, Germany).

For erythroblastic islet cell analysis, slides were fixed in pure methanol and stained with modified Giemsa solution (azure B/azure II, eosin, and methylene blue), according to the manufacturer’s protocol (Sigma-Aldrich, Taufkirchen, Germany). Cells were analyzed and counted in 100 randomly selected fields (0.01 mm^2^) using a light microscope at 1250× magnification. In total, at least 3000 cells were analyzed and classified at a given day before and after radiation.

### 4.5. Bone Marrow Sections

At least five samples from bone marrow (BM) were taken from each animal and fixed in 10% buffered formalin solution (pH 7.2) for 24 h. After fixation, the samples were dehydrated through a graded series of alcohols (70%, 80%, 96%, and 100%), washed with xylol, and embedded in paraffin wax. For morphological analysis, 5 μm wax-embedded samples were cut (Microm HM 355, Thermo Scientific, Waltham, MA, USA) and stained with routine methods using hematoxylin and eosin (HE). Histological examinations were undertaken using a light microscope (BOECO, BM-800, equipped with camera B-CAM10, Hamburg, Germany).

### 4.6. Hemoglobin Microspectrophotometry

Microspectrophotometry was performed using an SMP-05 Opton scanning microspectrophotometer to measure the spectra of hemoglobin (Hb); they were measured on air-dried smears of peripheral blood in unstained erythrocytes [72]. The spectrophotometric measurement was only performed at the wavelength of 416 nm for single RBCs, and the extracellular area was considered as a standard reference. The choice of microspectrophotometric method was due to the possibility of measuring small spectral changes with a limited number of RBCs [73]. Appropriate cytometric investigations were performed as well. Plasma hemoglobin determination was performed according to a method described by Soloni et al. [74].

### 4.7. DNA Image Scanning Cytometry

For image scanning cytometry and DNA measurement, air-dried smears of peripheral blood were fixed in 96% ethanol for 30 min and stained in fresh Schiff’s reagent (DNA hydrolysis in 5 N hydrochloric acid for 60 min at 22 °C) using the method developed by Feulgen [75,76]. The image scanning cytometry for the measurement of DNA content was done at a wavelength of 575 nm and magnification of 1250× using a computer-equipped microscope-cytometer SMP 05 (OPTON). The data were expressed in conventional units. Before the scanning process, each nucleus was contoured, and cytometry of the nuclear DNA content of all types of cells studied was carried out at 1 to 7 dpi.

### 4.8. Distribution of the Cells by Nuclear Ploidy Classes

DNA content was expressed on a “c” scale, in which 1 c is the haploid amount of nuclear DNA found in normal (non-pathologic) diploid populations in G0/G1. The DNA content of unstimulated rat lymphocytes was used as a diploid standard for measurements. DNA measurements identify nuclei as aneuploid if they deviate by more than 10% between 2 c, 4 c, and 8 c; i.e., if they are outside of the values of 2 c ± 0.2, 4 c ± 0.4, 8 c ± 0.8. The total numbers of cells in euploid areas of the DNA histogram rescaled by the mean corrective factor (1.8 c–2.2 c, 3.6 c–4.4 c, 7.2 c–8.8 c) were also calculated. The variability of the DNA content in unstimulated lymphocytes did not exceed 10% [77,78].

### 4.9. Determination of Hepatic Iron Accumulation

The hepatic iron accumulation was assessed in the parafinized liver sections using the method of Perl’s Prussian blue staining described earlier [79]. One percent aqueous neutral red was used as the counterstain.

### 4.10. Evaluations of Plasma Hemoglobin and Hematocrit

To study the plasma markers of blood hemolysis, the total plasma Hb concentration was measured by visible absorbance spectrophotometry [80]. Assays were performed in triplicate.

### 4.11. Determination of SOD and Catalase in Erythrocyte Hemolysates

The erythrocyte hemolysates were obtained from fresh whole-blood samples collected in EDTA-containing tubes to prevent coagulation, as described previously [81,82]. The hemolysate samples were stored at −30 °C until further use.

The activities of SOD and catalase in rat erythrocyte hemolysates were determined using the spectrophotometric methods described earlier [81,83,84,85], where the inhibition level of adrenaline auto-oxidation by SOD and the decrease of H_2_O_2_ content by catalase were detected at 374 nm and 420 nm, respectively.

### 4.12. Determination of Ceruloplasmin Ferroxidase Activity and Erythropoietin Levels in Plasma Samples

For the isolation of plasma samples, the blood samples collected in EDTA-containing tubes were kept on ice for 60 min [86], followed by centrifugation at 3000× *g* for 15 min at 4 °C; they were then put in storage at −30 °C until further use. Immediately prior to use, the plasma samples were thawed and centrifuged at 10,000× *g* rpm for 5 min at 4 °C to remove any precipitate.

The ferroxidase activity of ceruloplasmin was determined using the spectrophotometric method described earlier [81,87], where the level of the oxidation of the bivalent iron (contained in Mohr’s salt in a complex with o-phenanthroline) to the trivalent form (iron(III) ammonium sulfate) was detected at 420 nm (A420) using a microtiter plate reader (Stat Fax 3200, Awareness Technology Inc., Palm City, FL, USA).

The levels of erythropoietin were determined using commercially available ELISA kits (Bioassay Technology Laboratory, Shanghai, China) according to the manufacturers’ instructions. The concentrations of erythropoietin were expressed in pg per mL of plasma (pg/mL).

### 4.13. Determination of Oxidative Stress in Living Erythrocytes

The oxidative stress inside living RBCs was determined via the two-photon laser scanning microscopy imaging described earlier [81,82,88]. RBCs were isolated from the fresh blood samples using isotonic phosphate-buffered saline solution [81,82,88,89,90,91] and used for two-photon microscopy imaging immediately to avoid any alterations in the erythrocyte morphology. All the samples were treated with a membrane-permeable 5(6)-carboxy-2′,7′-dichlorofluorescein diacetate (carboxy-DCFDA, Sigma-Aldrich Chemie GmbH, Darmstadt, Germany) fluorescent dye [81,82,88,92,93,94,95]. Two-photon imaging was performed using the laser source available at the AREAL facility, which provides an excitation at the wavelength of 1030 nm and is attached to a two-photon laser-scanning upright microscope (Movable Objective Microscope (MOM), Sutter Instruments, Novato, CA, USA) [1,2,81,82,88].

### 4.14. Data Processing and Statistical Analysis

The images were processed using Fiji/ImageJ software (ImageJ 1.50 i NIH, Bethesda, MD, USA) [96], as described previously [81,82,88]. Statistical analysis was performed using GraphPad Prism 5.01 (GraphPad Software, San Diego, CA, USA). Depending on whether the data were parametric or non-parametric, one-way ANOVA or the Kruskal–Wallis tests were used, and the differences between groups were determined with Bonferroni or Dunn’s post hoc tests, respectively. Values are expressed as means ± standard error of the mean (SEM), and *p* < 0.05 was considered as the statistically significant value.

## 5. Conclusions

In conclusion, despite the fact that laser-driven UPEB irradiation requires quite low doses and repetition rates to achieve the LD_50_ in rats, our findings suggest that whole-body exposure with this new type of irradiation causes relatively mild anemia in rats, with subsequent fast recovery up to the 28th day. Moreover, this novel type of irradiation causes highly intense processes of oxidative stress, which, despite being relatively extinguished, do not reach a physiologically stable level even by the 28th day after irradiation. Finally, this new irradiation modality causes alterations in the antioxidant system as well; however, these alterations reach the normal physiological levels, which meant that it was not possible to quench the oxidative burst and maintain the redox balance even with trials in which the organism switched the available compensatory mechanisms in the antioxidant system. Although the alterations in the hematopoietic and oxidant/antioxidant systems induced by X-rays or gamma irradiation are well-known phenomena, the effects of laser-driven UPEB irradiation on these processes are here described for the first time.

## Data Availability

Not applicable.
